# Overcoming platinum resistance in ovarian cancer by targeting pregnancy-associated plasma protein-A

**DOI:** 10.1371/journal.pone.0224564

**Published:** 2019-11-21

**Authors:** Diogo Torres, Xiaonan Hou, Laurie Bale, Ethan P. Heinzen, Matthew J. Maurer, Valentina Zanfagnin, Ann L. Oberg, Cheryl Conover, S. John Weroha

**Affiliations:** 1 Department of Obstetrics and Gynecology, Division of Gynecologic Surgery, Mayo Clinic, Rochester, MN, United States; 2 Department of Oncology, Division of Medical Oncology, Mayo Clinic, Rochester, MN, United States; 3 Division of Endocrinology, Mayo Clinic, Rochester, MN, United States; 4 Department of Health Science Research, Division of Biomedical Statistics and Informatics, Rochester, MN, United States; Duke University School of Medicine, UNITED STATES

## Abstract

**Objectives:**

Inhibition of pregnancy-associated plasma protein-A (PAPP-A), an upstream activator of the insulin-like growth factor (IGF) pathway, is known to augment sensitivity to platinum-based chemotherapy. This study further tests the efficacy of PAPP-A inhibition with a monoclonal antibody inhibitor (mAb-PA) in ovarian cancer (OC) platinum-resistant patient-derived xenograft (PDX) models.

**Methods:**

PAPP-A expression was quantitated in platinum-resistant PDX models by ELISA. A subset with High (n = 5) and Low (n = 2) expression were revived in female SCID/beige mice for studies with either saline, carboplatin/paclitaxel (CP) + mAb-PA, or CP + IgG2a. The primary endpoint was tumor area by ultrasound on day 28 relative to baseline. Conversion to platinum-sensitive was defined by average tumor regression below baseline. Statistical analyses included linear mixed effects modeling and Kaplan Meier curves. Response to therapy was correlated with changes in the ratio of phosphorylated/total AKT and ERK 1/2 using Wes analysis.

**Results:**

The addition of mAb-PA to CP induced tumor regression below baseline in one High PAPP-A PDX model; another three models exhibited notable growth inhibition relative to CP + IgG2a. None of the Low PAPP-A PDX models regressed below baseline. The PDX model with the greatest magnitude of tumor regression from baseline after combination therapy was maintained on single agent mAb-PA or IgG2a, but no benefit was observed. Decreased phosphorylation of ERK1/2 correlated with conversion to platinum-sensitive.

**Conclusions:**

The addition of mAb-PA to CP overcame platinum-resistance in one of five High PAPP-A PDX models; three other models demonstrated improved platinum-response. This supports further clinical development of this novel therapeutic.

## Introduction

Front line treatment of ovarian cancer (OC) is a combination of surgery and platinum-based combination chemotherapy[1]. Recurrences are common and patients who recur >6 months after completion of primary therapy may benefit from repeat platinum-based chemotherapy. However, resistance to platinum chemotherapy will eventually occur[2] and standard salvage therapies have limited efficacy. Since OC is highly heterogeneous and high-grade serous OC rarely exhibits recurrent somatic mutations[3], therapies that target recurring oncogenic driver mutations are less likely to have a significant impact on this disease. However, an alternative approach to overcome platinum resistance is to target dysregulated pathways contributing to pro-survival and anti-apoptosis signaling.

**Fig 1:**
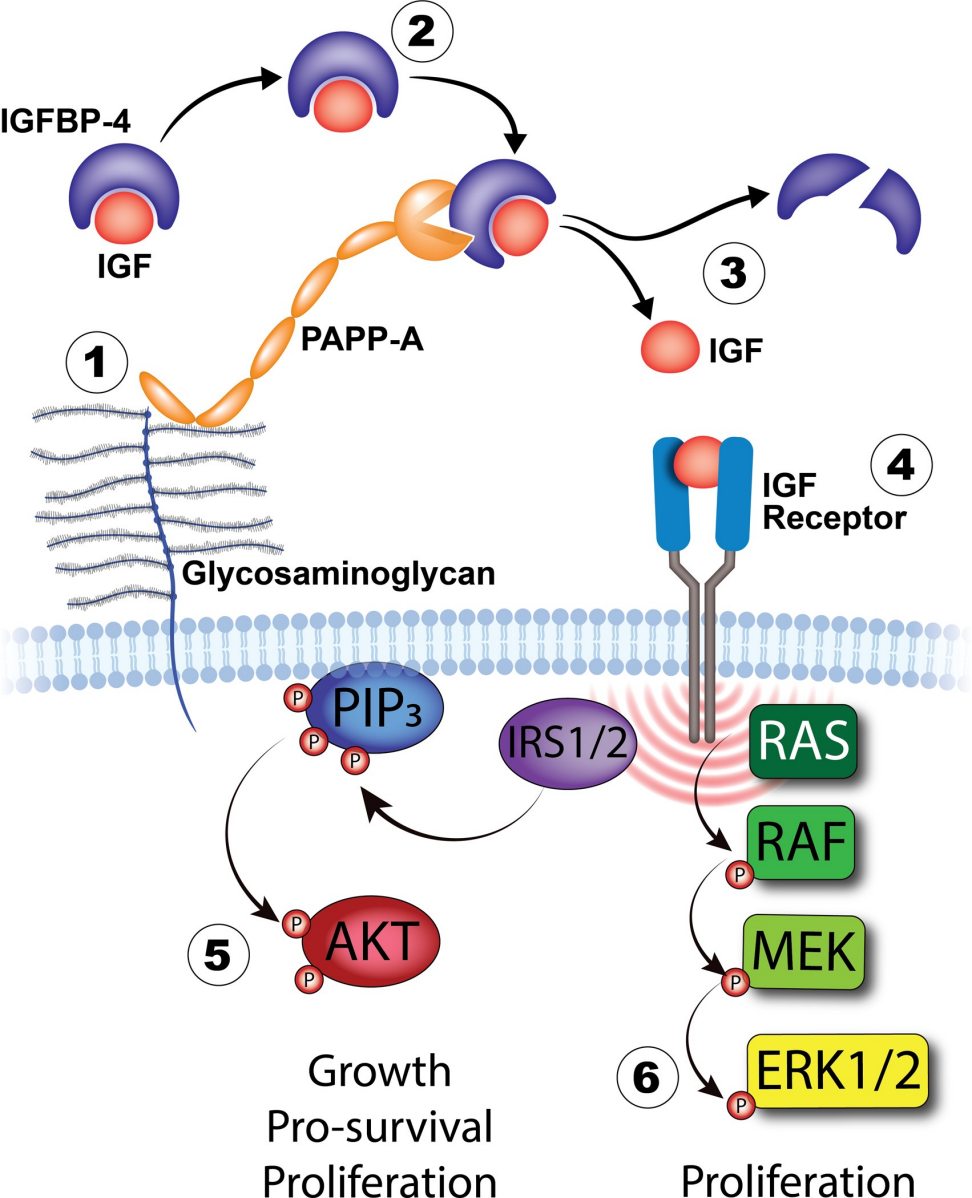
Local control of IGF signaling by cell-associated PAPP-A. (1) PAPP-A is a secreted enzyme that associates with heparin-like proteoglycans on the surface of the secreting and neighboring cells through the third and fourth of five C-terminal short consensus repeats. This tethering localizes the effects of PAPP-A. (2) IGFBP-4 binds IGF with high affinity and sequesters the ligand from interacting with cell surface receptors. (3) PAPP-A cleaves IGFBP-4 in an IGF-dependent manner (e.g. only when IGF is bound to IGFBP-4), markedly reducing the affinity of IGFBP-4 to IGF. (4) IGF is released into the pericellular environment, facilitating receptor binding. IGF binding initiates specific IGF-1 receptor signal transduction through AKT (5) or RAS/ERK (6) to mediate mitogenesis, metabolic effects, and/or survival under tissue- and context-specific conditions.

The insulin-like growth factor (IGF) system is a signaling pathway that plays an important role in tumorigenesis and is a potential therapeutic target (Fig 1)[4]. Activation of the insulin-like growth factor 1 (IGF-1R) pathway has been associated with several malignancies, including ovarian cancer[4], by inducing cellular proliferation and survival through upregulation of the PI3K-AKT, MAPK/ERK, and IRS2 pathways[5–7]. Multiple clinical studies have investigated the efficacy of targeting the IGF pathway with a monoclonal antibody (mAb) against IGF-1R (mAb-IGF-1R), primarily in patients with breast, lung, or sarcoma cancer[8–12]. Other strategies have targeted the IGF ligand, such as monoclonal antibodies to IGF-1/2, or inhibited the intracellular component of IGF-1R[13, 14]. Although a lack of substantial single-agent activity has limited further clinical development to this therapeutic approach, the efficacy of IGF pathway inhibition in combination with chemotherapy is understudied. Moreover, a reliable biomarker of response has not been identified; IGF-1R expression alone is insufficient [8]. Therefore, further studies are warranted to investigate novel biomarkers of response to inhibition of the IGF system.

An alternative approach to IGF targeting involves modulation of IGF binding proteins (IGFBPs). IGFBPs downregulate IGF-1R activity by decreasing the extracellular availability of unbound ligand[15]. In contrast, pregnancy-associated plasma protein-A (PAPP-A), a cell membrane-associated protease, cleaves and inactivates IGFBP-4 and -5 to increase the local concentration of IGF ligand available to bind to IGF-1R [16, 17]. Expression levels of PAPP-A vary from non-detectable to relatively high in OC [18]. Overexpression of PAPP-A in OC cell lines triggers tumorigenesis and cellular invasion; SKOV-3 cell lines transfected with a wild-type PAPP-A vector demonstrated higher cellular growth in soft agar gels and higher cell counts in chamber assay membranes compared to cells transfected with an empty vector[19].

A barrier to the further study of the IGF-1 pathway and PAPP-A inhibition in OC has been the paucity of clinically-relevant models. OC cell lines are prone to genetic drift with genotypic and phenotypic divergence from the original tumor occurring over time [20]. However, primary patient-derived xenograft (PDX) OC models recapitulate key characteristics of the primary patient tumor and enable the study of combinations and search for biomarkers that may ultimately lead to better outcomes in OC [21–23]. Importantly, PDX tumors exhibit a comparable treatment response phenotype to Carboplatin/Paclitaxel (CP) [23], underscoring the utility of using PDX models to study the effects of PAPP-A targeting in OC.

Preliminary studies have demonstrated that targeting PAPP-A may overcome platinum resistance [24]. A high-affinity IgG monoclonal antibody against PAPP-A (mAb-PA) selectively targets a unique exosite on PAPP-A which is required for proteolysis of IGFBP-4[25]. In a single platinum-resistant OC PDX model with relatively high PAPP-A expression, treatment with mAb-PA was safe and when combined with CP enhanced the efficacy of chemotherapy and induced tumor regression below baseline (i.e. a characteristic of a platinum-sensitive OC) [24]. Although this observation was interesting and raises the possibility that PAPP-A inhibition might help overcome platinum-resistance, confirmation of this observation is needed across additional models. In addition, a biomarker of response would be helpful to facilitate stratification of patients on future clinical trials.

The objective of this study was to confirm the therapeutic activity of mAb-PA and test the hypothesis that the combination of CP and mAb-PA will improve the efficacy of CP in a panel of OC PDX models that express PAPP-A. Positive results may help identify a predictive biomarker for eventual translation to clinical trials in patients with platinum-resistant OC.

## Methods

### Anti-PAPP-A monoclonal antibody

The development and characterization of the mAb-PA antibody and its effectiveness in inhibiting PDX tumor growth by inhibiting IGFBP-4 proteolysis have been reported previously[17]. It is a high-affinity murine IgG monoclonal antibody that recognizes the substrate-binding exosite (C-terminally located LNR3 site) of PAPP-A involved in lysis of the IGFBP-4/IGF-1, 2 ligand complex[25]. Intraperitoneal delivery in PDX mouse models of 30 mg/kg was safe and tolerable in preliminary studies[17, 24]. Monoclonal antibody to PAPP-A was provided by Ansh Labs, Webster, TX.

### Establishment of patient-derived tumor xenograft (PDX) models

After approval by the Mayo Clinic Institutional Review Board (IRB), fresh tissue from consenting patients with ovarian, primary peritoneal or fallopian tube cancer was obtained at the time of primary debulking surgery and immediately processed in the research laboratory. A unique identifier [patient heterotransplant (PH) number] was issued in accordance with the Health Insurance Portability and Accountability Act. Prior to injection, primary tumor specimens were minced and mixed with McCoy’s media containing 10 mg/kg of rituximab (Rituxan; Genentech, Inc; San Francisco, CA) to prevent human lymphoproliferation[26]. Minced tissue pieces (0.1cm^3^ per mouse) were injected intraperitoneally into at least 3 female SCID-bg mice (C.B.-17/IcrHsd-Prkdc^*scid*^ Lyst^bg^; ENVIGO), in accordance with the Mayo Clinic Institutional Animal Care and Use Committee (IACUC). Upon engraftment, tumor was collected and cryopreserved.

### Human PAPP-A detection by ELISA assays

PDX tumor samples were snap frozen in liquid nitrogen and then pulverized using the Cellcrusher (Cork, Ireland). Protein lysates were prepared in M-PER extraction buffer (Thermo Scientific, Waltham, MA) containing a protease inhibitor cocktail (Cat#P-8340, Sigma-Aldrich, St. Louis, MO) and NP40 cell lysis buffer. PAPP-A ELISA kits were generously provided by Ansh Labs (Cat#AL-101; Webster, TX), and were used to quantitatively measure human PAPP-A protein expression. This assay does not recognize murine PAPP-A[24]. Tumors were ranked per PAPP-A expression and split evenly into two cohorts, defining the top 50% as “High PAPP-A” and the bottom 50% as “Low PAPP-A.

### Defining platinum resistant OC PDX models

Platinum resistance was defined by a tumor fold change above baseline after four weeks of treatment with IP Carboplatin (APP Pharmaceuticals, Lake Zurich, Illinois)/Paclitaxel (Actavis Pharma, Inc.) at 50 and 15 mg/kg, respectively, on days 1, 8, 15, and 22 as described[23, 27].

### *In vivo* efficacy studies with mAb-PA

In accordance with the Mayo Clinic IACUC guidelines, six to eight week old severe combined immunodeficient (SCID) beige, female mice (C.B.-17/IcrHsd-Prkdc^scid^ Lyst^bg-J^; Envigo, Indianapolis, IN, USA) were injected intraperitoneally with primary platinum-resistant OC patient tissue prepared as previously described[23, 26]. Briefly, cryopreserved PDX tumor was rapidly thawed, and 0.1–0.2 cc of minced tumor slurry was prepared 1:1 in McCoy’s media. Tumor implantation was by intraperitoneal injection through a 16 gauge needle. Upon engraftment, tumor was expanded into 34–45 mice and monitored until they reached ≥0.2 cm^2^ by ultrasonography (Fujifilm SonoSite S-Series with on-board measurement tools). Mice were then randomized to either IP (A) saline (n = 4 to 6), (B) CP + mAb-PA (n = 15 to 20), or (C) CP + murine IgG2a (n = 13 to 21) (Cat#BE0085; Bio x Cell, West Lebanon, NH) on days 1, 8, 15, and 22. Tumors were measured weekly by transabdominal ultrasounds and the sonographer was blinded to treatment arms. Change in tumor area on day 28 vs. 0 was the primary endpoint. To ensure the final tumor change was not influenced by variability in initial tumor size, initial tumor size was included as a randomization criterion. Tumors that persisted unchanged or progressed above baseline on day 28 were defined to have remained platinum-resistant. Tumors that regressed below baseline on day 28 were defined to have platinum-sensitive conversion (i.e. induced platinum-sensitivity in an otherwise platinum-resistant tumor). After 28 days, the primary study ended and all mice in cohort A, and three each in cohorts B and C were euthanized. The remaining mice entered the secondary study to evaluate the therapeutic impact of maintenance mAb-PA. Mice in the secondary study that received mAb-PA in the primary study were randomized to receive continued mAb-PA or switched to IgG2a. Disease burden was assessed by weekly ultrasound for up to 12 weeks. Progression was defined as a threefold or higher increase in tumor area from day 28 or moribund, including tumor burden. Humane endpoints for removal of an animal before the planned experimental endpoints were: weight loss greater than or equal to 20% of body weight, inability to ambulate, inability to reach for food and/or water, tumors greater than or equal to 10% body weight, tumors that have ulcerated, a body condition score of 1 or less using the IACUC approved scoring system. The number of animals meeting moribund endpoints is detailed in the S1 Fig.

### Assessment of intratumor mAb-PA perfusion

Five μm cryosections of tumors treated *in vivo* with saline and CP + mAb-PA were harvested from mice, fixed in cold 4% paraformaldehyde solution for 15 min, air dried on glass slides, and stored at -80°C. To detect the presence of mAb-PA in tumors, sections were rehydrated in phosphate-buffered saline and blocked in protein block serum-free buffer (Cat#X0909; Dako, Carpinteira, CA) for 30 minutes at room temperature. Sections were subsequently incubated with a FITC-labeled anti-mouse IgG2a (Alexa Fluor® 568 goal; Life Technologies, Waltham, MA) at 1:1500 for 60 minutes at room temperature. Tumors treated with saline were an appropriate negative control due to SCID not having mature B cells. Slides were mounted with VECTASHIELD Antifade Mounting medium with DAPI (Cat# H-1500; Vector Laboratories; Burlingame, CA) and examined by immunofluorescence microscopy on a Zeiss Axiovert with a N.A. 1.40 63 × lens and photographed on a Zeiss Axiocam MRm CCD camera using Zeiss Zen software.

### Expression of phosphorylated and total AKT and ERK1/2

Wes-Rabbit (12–230 kDa) Master Kit (#PS-MK01) including anti-rabbit secondary antibody, antibody diluent, molecular weight ladder, streptavidin-HRP, dithiothreitol (DTT), fluorescent master mix, luminol-S, peroxide, sample buffer, and wash buffer were purchased from ProteinSimple (San Jose, CA, USA). Capillary Western analyses were performed using the ProteinSimple Wes System. Sample replicates (whole-tissue extracts) from a given treatment group were pooled and diluted with 0.1 × Sample Buffer. Then 4 parts of diluted sample were combined with 1 part 5 × Fluorescent Master Mix (containing 5 × sample buffer, 5 × fluorescent standard, and 200 mM DTT) and heated at 95°C for 5 min. The Fluorescent Master Mix contained three fluorescent proteins as intra-capillary reference standards in addition to a separate capillary containing the same reference proteins. A biotinylated ladder provided molecular weight standards for each assay. The separation electrophoresis and immunodetection steps took place in the fully automated capillary system. The Wes System was programmed to add blocking reagent, primary antibody (1:100 dilution), HRP-conjugated secondary antibody, and chemiluminescent substrate. Antibodies used were specific for total ERK1/2 (Cat#9102), pERK1/2 (Cat#4377), total AKT (Cat#9272), and pAKT^S473^ (#4060) (Cell Signaling, Danvers, MA). Densitometric analysis was performed in Wes System for total ERK1/2, pERK1/2, total AKT, and pAKT on pooled protein extracts from three tumors within the same cohort to assess the average expression.

### Statistical analysis

A repeated measures analysis using data from all time points was used to compare tumor growth trajectories between study groups within each PDX model as done previously[21]. This is more powerful and less subject to type I error than performing t-tests at each time point [28–31]. Technically, this was carried out using a linear mixed effects modeling framework as described in the S1 Supplementary Statistical Methods. Unlike repeated measures ANOVA in which mice are excluded if they must be sacrificed prior to the end of the study, this modeling framework allows all mice to be used in the analysis regardless of the amount of follow up. This also enables more stable estimates of error bars (shown graphically herein as shadowed 95% confidence limits) over the full length of follow up, even when the number of mice remaining is small. A single test of coincident curves (accounts for both mean and slope) was performed for each pair of treatment arms. Progression free survival (PFS) was compared between treatment groups using Kaplan-Meier and log-rank test methods. Analyses were performed via SAS software. Copyright, SAS Institute Inc. SAS and all other SAS Institute Inc. product or service names are registered trademarks or trademarks of SAS Institute Inc., Cary, NC, USA.

## Results

### Wide range of PAPP-A expression in platinum-resistant PDX models

To help identify appropriate models for testing, an ELISA assay was used to quantify PAPP-A expression in 19 platinum-resistant PDX models (key characteristics are described in S1 Table, as recommended by guidelines [32]). PAPP-A concentration ranged from undetectable to 1.3 ng PAPP-A/mg of total protein (S2 Table). PAPP-A levels were ordered from highest to lowest and split evenly into two cohorts: the top 50%ile as PAPP-A “High” and the bottom 50%ile as PAPP-A “Low.” Five High (PH271, PH358, PH471, PH006, and PH231) and two Low (PH386, and PH450) PDX models were selected (S2 Fig).

### mAb-PA combined with platinum-based chemotherapy improved platinum response

To determine the ability of mAb-PA to improve platinum response in otherwise platinum- resistant PDX models, seven platinum-resistant PDX models were reconstituted in female SCID-beige mice by IP injection following standard procedures[23]. Mice were then randomized to receive either IP saline, CP + mAb-PA, or CP + IgG2a (control antibody) for 28 days. Tumors that persisted unchanged or progressed above baseline at the end of CP + mAb-PA treatment were defined as remaining platinum-resistant. Tumors that regressed below baseline at the end of CP + mAb-PA treatment converted to platinum-sensitive. Individual (per mice) growth curves and the relative number of mice that reached the 28 days endpoint are provided in S1 Fig.

**Fig 2:**
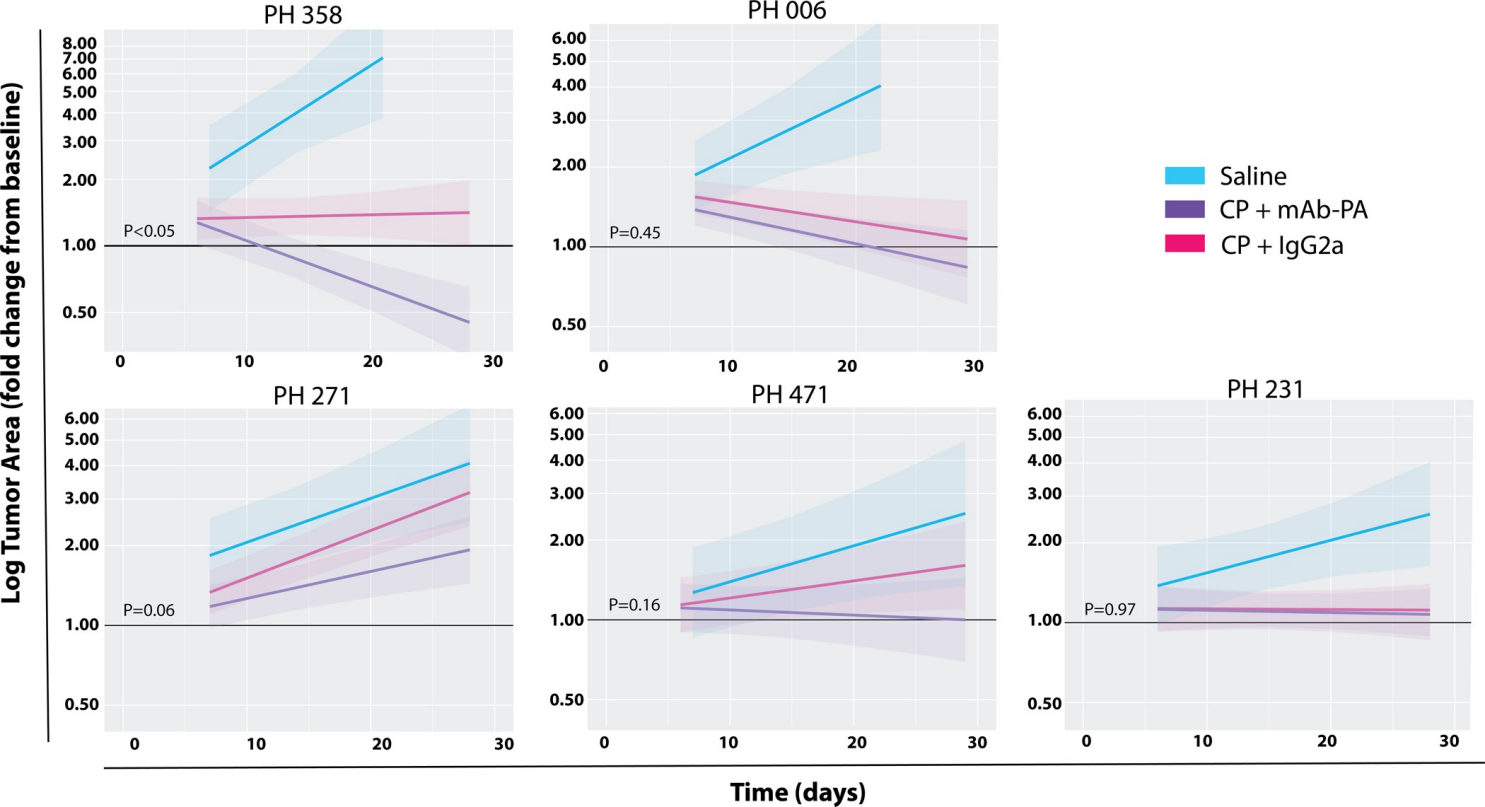
Ovarian cancer PDX *in vivo* response to therapy in five high PAPP-A models. Model averages with 95% confidence intervals (shaded area) are plotted on the fold change from baseline scale as a function of time. Platinum response was defined as average tumor area (cm^2^) regression below baseline (day 0) on day 28 in response to Carboplatin/Paclitaxel (CP) or the combination of CP and monoclonal antibody against PAPP-A (mAb-PA). The control antibody (IgG2a) was isotype matched. P-values correspond to a coincident curve test, which tests for differences in mean, slope, or both.

Initial analyses were performed on the five High PAPP-A models (Fig 2). All models treated with CP + IgG2a met the definition for platinum-resistance, including PH006 (despite the downward slopping trajectory approaching baseline). Only one model (PH358) exhibited significantly different tumor size trajectories across both arms (CP + IgG2a vs CP + mAb-PA, p = 0.0002). Strikingly, 14 of 19 (74%) tumors treated with CP + mAb-PA regressed below baseline with an average tumor regression of 55% (95% CI: 35–69%) below baseline. Although the tumor size trajectories in the combination group (CP + mAb-PA) for four other models (PH006, PH271, PH231, and PH471) were not significantly different from the CP + IgG2a group, all tended to have smaller tumors after combination therapy, except for PH231, in which trajectories are nearly overlaid. Since a goal of this study was to sensitize tumors to platinum-based therapy, models were observed for conversion from platinum-resistant to platinum-sensitive, based on the pre-determined criterion of regression below baseline. In this regard, average trajectories for two models (PH358 and PH006) met criterion for conversion to platinum-sensitive. However, the response in PH006 was less pronounced than PH358 with only 8 of 19 (42%) tumors regressed below baseline and the two growth trajectories (CP + mAb-PA and CP + IgG2a) did not differ significantly(p = 0.45). Model PH231 did not demonstrate a difference in tumor growth across treatment arms (p = 0.97). Collectively, these results indicate that one of the five High PAPP-A models converted to platinum-sensitivity with combination therapy and three others benefited qualitatively.

**Fig 3:**
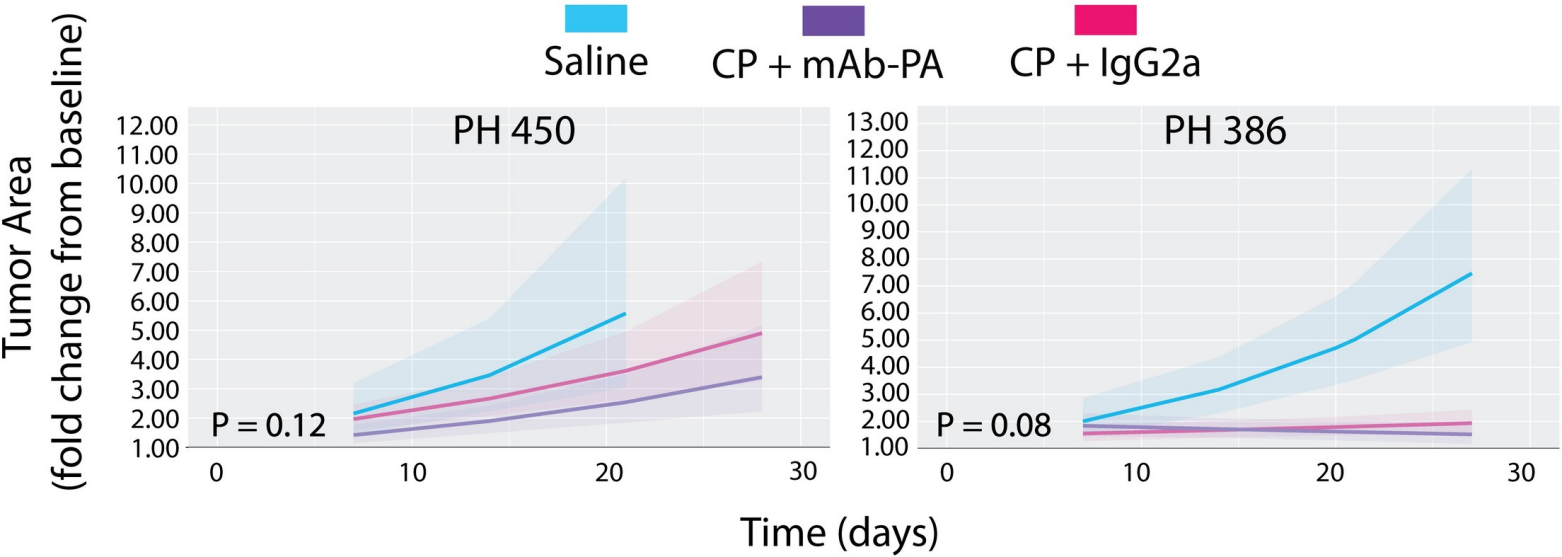
Ovarian cancer PDX *in vivo* response to therapy in two low PAPP-A models. Model averages with 95% confidence intervals (shaded area) are plotted on the fold change from baseline scale as a function of time. Platinum response was defined as average tumor area (cm^2^) regression below baseline (day 0) on day 28 in response to Carboplatin/Paclitaxel (CP) or the combination of CP and monoclonal antibody against PAPP-A (mAb-PA). The control antibody (IgG2a) was isotype matched. P-values correspond to a coincident curve test, which tests for differences in mean, slope, or both.

None of the two low PAPP-A models regressed below baseline with combination therapy on average, but tumor growth was attenuated in PH450 (though not significant) compared to the CP + IgG2a group (Fig 3). Although the growth trajectories crossed in model PH386, the trajectories were not significantly different (p = 0.08).

### Similar intra-tumor perfusion of mAb-PA antibody across PDX models

To ensure that the lack of platinum-sensitive conversion in High PAPP-A models was not due to poor antibody infiltration into the tumor, post-treatment tumors from mice in the combination therapy arm and saline arm were frozen to preserve any intra-tumor immunoglobulins. Cryosections were then exposed to fluorescently labeled anti-mouse antibodies recognizing IgG2a (the mAb-PA isotype). Positive staining for IgG2a indicated the presence of mAb-PA in High and Low PAPP-A models (S3 Fig). Signal intensity and distribution was comparable between PDX models. No IgG was detected in saline controls, as expected for SCID mice lacking mature B cells.

### No appreciable benefit to maintenance therapy with mAb-PA

**Fig 4:**
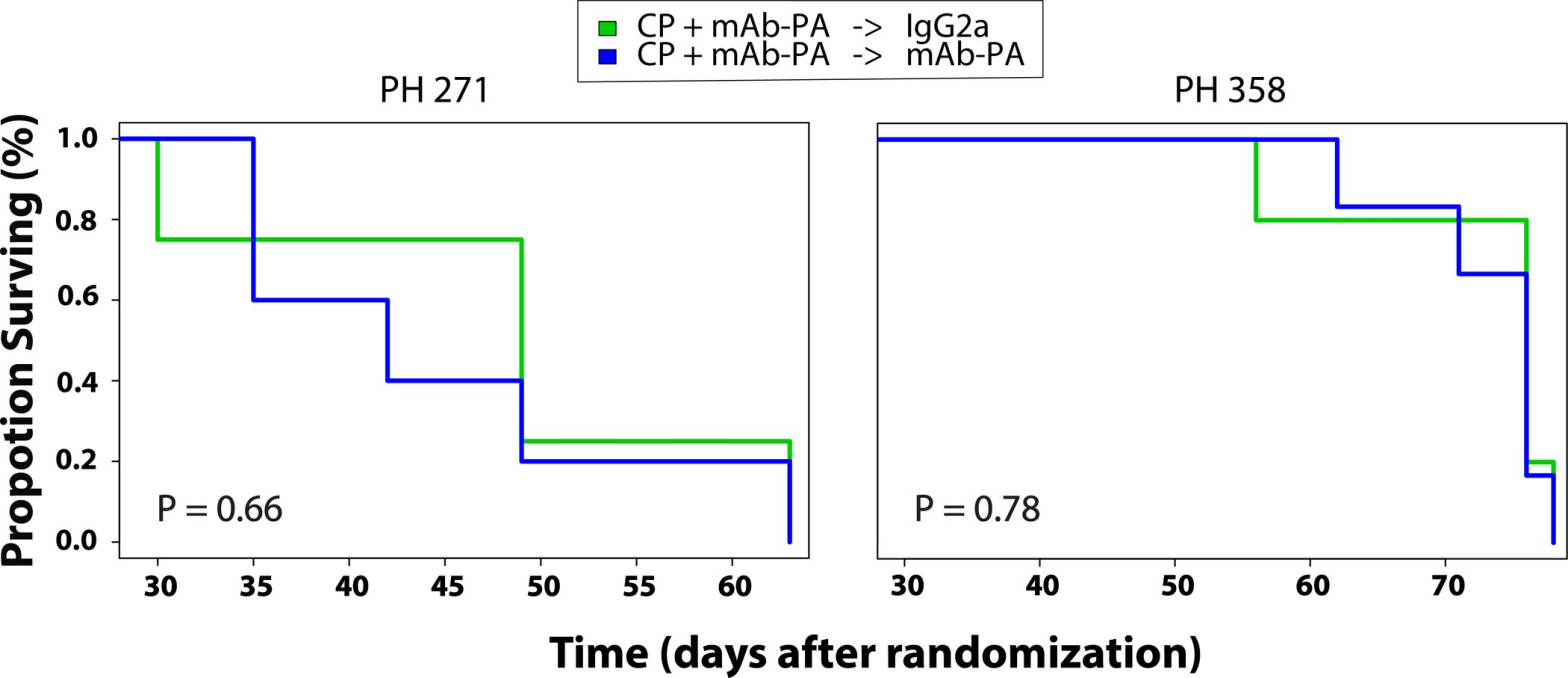
Progression-free survival (PFS) in two high PAPP-A models. Prior to randomization into the secondary study, models were treated with Carboplatin/Paclitaxel (CP) and mAb-PA. Mice were maintained on either control antibody (IgG2a) or monoclonal antibody against PAPP-A (mAb-PA).

The secondary objective was to assess if maintenance mAb-PA therapy augmented response to CP or delayed progression of disease in High PAPP-A models. Since maintenance therapy is mostly likely to show benefit if there is an initial response to mAb-PA, model PH358 was shown as the best responder and PH271 was included as a reference model since it did not regress below baseline. Mice treated with saline or control antibody in the primary study were excluded. Only a subset of mice treated with CP + mAb-PA were randomized to maintenance therapy with either control antibody or mAb-PA. No benefit in PFS was observed in either model randomized to maintenance mAb-PA versus control antibody (PH358, p = 0.78; PH271 p = 0.66) (Fig 4).

### Downstream targets of IGF-1R are inhibited by mAb-PA

**Fig 5:**
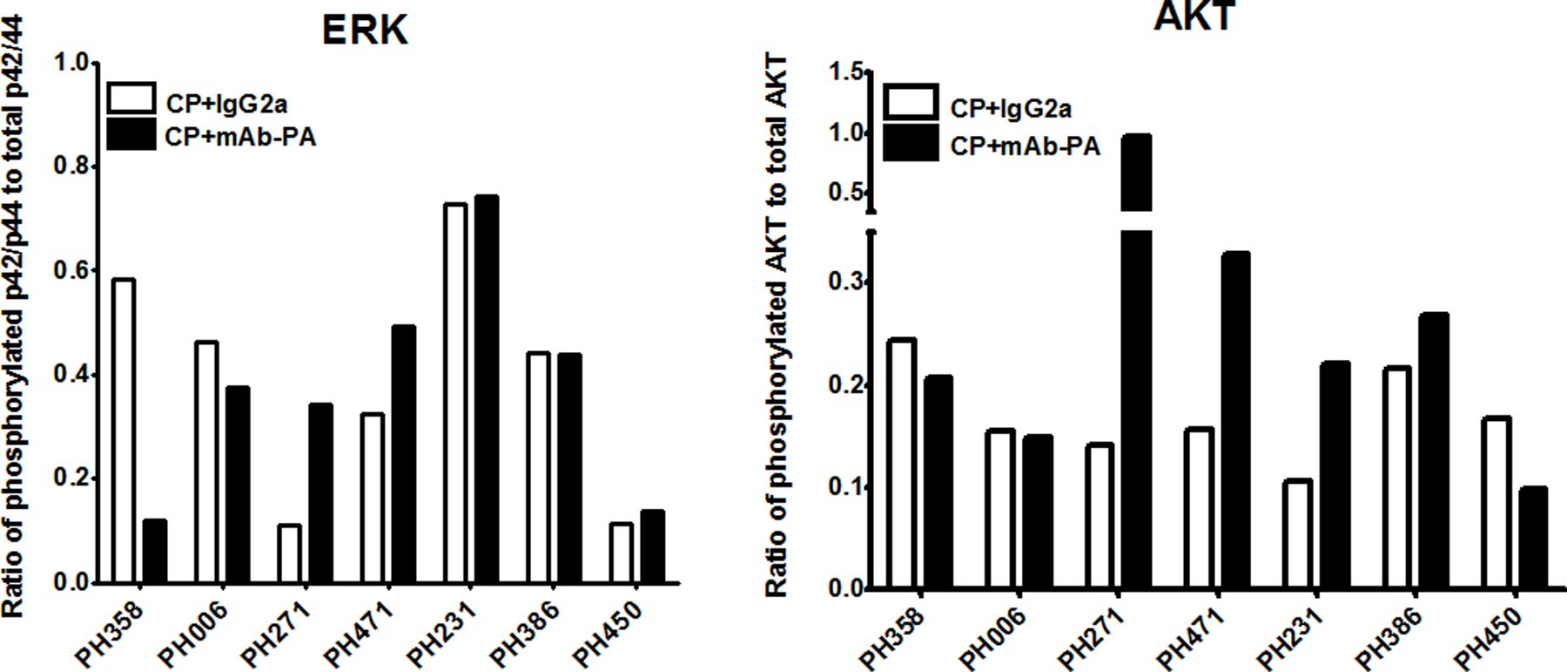
Changes in phosphorylated and total proteins downstreat of IGF-1R. [A] The ratio of phosphorylated p42/p44 (ERK 1/2) to total p42/p44 and [B] ratio of phosphorylated to total AKT protein expression in seven representative PDX models is shown after treatment with saline, Carboplatin/Paclitaxel (CP) plus monoclonal antibody against PAPP-A (mAb-PA), or control antibody (IgG2a). For each PDX model, four biological replicates from a given treatment group were pooled in equal parts to provide an average.

On-target activity of mAb-PA was assessed to investigate the purported mechanism of action. Direct assessment of IGF-1R expression is an unreliable measure of pathway activity. Alternatively, phosphorylation of IGF-1R pathway proteins, such as AKT and ERK, has been used to demonstrate pathway activation [5–7] since activated IGF-1R promotes phosphorylation of AKT and ERK 1/2. Inhibition of the IGF pathway was expected to decrease phosphorylation of AKT and ERK 1/2[18]. Accordingly, capillary Western blot (Wes) analysis was used to measure levels of phosphorylated and total protein in selected PDX models as a measure of IGF pathway activity (Fig 5). Seven of eight representative models with varying degrees of response to therapy were chosen. A marked decrease in phosphorylated ERK 1/2 (normalized to total levels) following treatment with mAb-PA was only observed in model PH358, consistent with the PDX response. However, similar changes were not seen with phosphorylated AKT in PH358, suggesting the ERK pathway may play a more dominant role in mAb-PA therapy and suggesting that a decrease in phosphorylated ERK 1/2 was associated with conversion to platinum-sensitive while phosphorylated AKT was not.

## Discussion

The addition of a novel neutralizing antibody against PAPP-A (mAb-PA) to CP improved the outcome for a subset of OC PDX models with relatively high levels of PAPP-A expression. Combination therapy overcame platinum-resistance in 20% (1/5; PH358) of platinum-resistant OC PDX models and 60% (3/5; PH006, PH271, and PH471) had a trend towards benefit. None of the two Low PAPP-A PDX converted to platinum-resistant. Maintenance therapy with mAb-PA did not improve PFS. Decreased phosphorylation of ERK correlated with conversion to platinum-sensitive. These results suggest that PAPP-A inhibition converted one platinum-resistant PDX model to platinum-sensitive via the IGF pathway and supports the rationale for using PAPP-A protein expression to enrich for tumors with greatest likelihood of benefit from mAb-PA treatment.

Although studies combining chemotherapy and IGF-1R inhibitors have been reported using OC cell line xenografts *in vivo*[33–35], the current study is the largest to examine the efficacy of CP with an IGF-1R pathway inhibitor in clinically relevant platinum-resistant OC PDX models. A smaller study combined IGF-1R inhibitor BMS-754807 with CP in a carcinosarcoma OC PDX (PH003) but failed to show a therapeutic benefit, possibly due to differences in drug mechanism and selection criteria [22]. Importantly, this current study is consistent with the observation that mAb-PA can partially overcome platinum resistance *in vivo* [24] and goes a step further by expanding the cohort to eight OC PDX models with various histology (serous, carcinosarcoma, clear cell, and squamous/transitional) to better define the efficacy of mAb-PA. Moreover, assessment of the downstream targets of IGF-1R suggests that tumor regression may be dependent on the inhibition of ERK signaling.

PDX models that persist or progress above baseline after 28 days of treatment with CP are defined as platinum-resistant; those that regress below baseline are defined as platinum-sensitive. These definitions are based on a previous study that demonstrated a strong correlation between response to CP in PDX models and the matched patient’s clinical response to platinum chemotherapy, supporting the relevance of this definition [23]. For example, patient tumors that were clinically defined as platinum-sensitive were associated with PDX tumors that regressed below baseline after 28 days of treatment. All PDX models in this study were platinum-resistant. Model PH006 did have a trajectory towards platinum-sensitive with CP + IgG2a but it did not fall below baseline after 28 days of treatment (Fig 2); therefore, it meets the definition of platinum-resistant. Although it is recognized that a longer study duration may have led to regression of PH006 below baseline for this arm, pre-study definitions were upheld. Furthermore, model PH006 with CP + mAb-PA did regress below baseline after 28 days of treatment. However, it did not exhibit a statistically significant difference between CP + mAb-PA and CP + IgG2a treatments. Therefore, PH006 is considered to have benefited from therapy.

Since mAb-PA appears to improve the efficacy of CP in platinum-resistant ovarian cancer, it could play an important role in front line therapy. TRIO-014 failed to demonstrate a benefit in PFS by adding anti-IGF-1R antibody (AMG 479, ganitumab) to combination chemotherapy in patients with a new diagnosis of OC but did not include a biomarker-based inclusion criterion [36]. In addition, it did not include the patients at highest risk for recurrence by including only patients with optimally debulked disease. Accordingly, a larger sample size may be needed to appreciate an improvement in PFS in such patients. A potential development strategy for mAb-PA might be to target patients with suboptimally debulked disease since patients with residual disease > 1cm have worse survival outcomes[37]. This approach would typically provide ample tissue and time to measure PAPP-A levels by ELISA for inclusion. Although TRIO-014 excluded carcinosarcomas, the data presented herein supports a histology-agnostic approach as demonstrated by the qualitatively improved response seen in PH006.

In conclusion, novel therapies are needed to improve clinical outcomes in patients with platinum-resistant OC. The recognition that *in vitro* models of cancer can be molecularly divergent from the patient’s primary OC [20] has stimulated growing utilization of clinically relevant PDX models for preclinical drug development. The experiments presented herein demonstrate the potential value of PDX models in this regard and suggest that screening for PAPP-A levels with an ELISA assay can potentially select for a subgroup of platinum-resistant OC tumors most likely to benefit from PAPP-A inhibition and potentially conversion to platinum-sensitive.

## Supporting information

S1 Supplementary statistical methods Further technical details regarding the mixed effects models include that the response variable was change in tumor area from baseline on the natural log scale[21]. Thus, exponential growth is assumed. Predictor variables included time, study arm, and the interaction between time and study arm. The time variable was centered for hypothesis testing, and thus can be interpreted as the overall average or an area under the curve[28–31]. The slope is interpreted as a rate of growth. The intercept and slope were specified as random effects with unstructured correlation, allowing per-mouse regression lines. Because of occasional differences in measurement intervals, a spatial power correlation structure was used, which assumes any two observations from the same mouse are correlated and that this correlation decreases exponentially with time between the observations. For visualization, model estimates with 95% confidence intervals were plotted for each treatment group and are displayed as shadows. A two degree of freedom test of coincident curves utilized data after baseline through day 28 to compare growth rates between treatment arms. The null hypothesis is that the growth curves are coincident, i.e., have the same intercept (mean) and slope. The alternative hypothesis is that the growth curves differ in intercept, slope or both. Average results are based on model predicted values.
(DOCX)

S1 Fig Ovarian cancer PDX *in vivo* response to therapy in high and low PAPP-A models. Dashed lines are individual mouse tumor area trajectories as a function of time on the fold change from baseline scale. Solid lines with shading are model predicted values with 95% confidence intervals. Numbers below the x-axis indicate number of mice still being followed at each time point for each treatment group.
(TIF)

S2 Fig PDX models ranked from highest to lowest PAPP-A levels. Red arrows represent high PAPP-A models selected for study. Green arrows represent low PAPP-A selected for study.
(TIF)

S3 Fig Immunofluorescent staining of tumor tissues showing penetration of monoclonal antibody against PAPP-A (mAb-PA), regardless of response to therapy. Post-treated samples from a saline control (left) and Carboplatin/Paclitaxel (CP) plus mAb-PA (right) were probed with a poly-clonal anti-mouse antibody to detect presence of mAb-PA or background mouse IgG. A high PAPP-A model (PH358), which regressed below baseline when treated with CP + mAb-PA, show no background mouse IgG [A] and positive staining (red) for mAb-PA intratumor penetration [B]. A similar pattern was observed with PH271 [C and D], which did regress below baseline when treated with CP + mAb-PA. Tumors treated with CP + IgG2a had similar immunofluorescent staining patterns to panels [B] and [D] (not shown). DAPI was used to stain nuclei (blue).
(TIF)

S1 Table PDX models minimal information standard (PDX-MI). (DOCX)

S2 Table Range of PAPP-A concentration (ng). (DOCX)
