# Supplementary material for: Overcoming platinum resistance in ovarian cancer by targeting pregnancy-associated plasma protein-A
Source: PLoS One. 2019 Nov 21;14(11):e0224564. doi: 10.1371/journal.pone.0224564 (PMC6872139; doi:10.1371/journal.pone.0224564)

**SUPPLEMENTARY**

**Supplementary Statistical Methods.** Further technical details regarding the mixed effects models include that the response variable was change in tumor area from baseline on the natural log scale([21](#_ENREF_21)). Thus, exponential growth is assumed. Predictor variables included time, study arm, and the interaction between time and study arm. The time variable was centered for hypothesis testing, and thus can be interpreted as the overall average or an area under the curve([28-31](#_ENREF_28)). The slope is interpreted as a rate of growth. The intercept and slope were specified as random effects with unstructured correlation, allowing per-mouse regression lines. Because of occasional differences in measurement intervals, a spatial power correlation structure was used, which assumes any two observations from the same mouse are correlated and that this correlation decreases exponentially with time between the observations. For visualization, model estimates with 95% confidence intervals were plotted for each treatment group and are displayed as shadows. A two degree of freedom test of coincident curves utilized data after baseline through day 28 to compare growth rates between treatment arms. The null hypothesis is that the growth curves are coincident, i.e., have the same intercept (mean) and slope. The alternative hypothesis is that the growth curves differ in intercept, slope or both. Average results are based on model predicted values.

**S1 Fig. Ovarian cancer PDX *in vivo* response to therapy in High and Low PAPP-A models.** Dashed lines are individual mouse tumor area trajectories as a function of time on the fold change from baseline scale. Solid lines with shading are model predicted values with 95% confidence intervals. Numbers below the x-axis indicate number of mice still being followed at each time point for each treatment group.

**S2 Fig.** **PDX models ranked from highest to lowest PAPP-A levels.** Red arrows represent high PAPP-A models selected for study. Green arrows represent low PAPP-A selected for study.

**S3 Fig.** **Immunofluorescent staining of tumor tissues showing penetration of monoclonal antibody against PAPP-A (mAb-PA), regardless of response to therapy.** Post-treated samples from a saline control (left) and Carboplatin/Paclitaxel (CP) plus mAb-PA (right) were probed with a poly-clonal anti-mouse antibody to detect presence of mAb-PA or background mouse IgG. A high PAPP-A model (PH358), which regressed below baseline when treated with CP + mAb-PA, show no background mouse IgG [A] and positive staining (red) for mAb-PA intratumor penetration [B]. A similar pattern was observed with PH271 [C and D], which did regress below baseline when treated with CP + mAb-PA. Tumors treated with CP + IgG2a had similar immunofluorescent staining patterns to panels [B] and [D] (not shown). DAPI was used to stain nuclei (blue).

**S1 Table**. **PDX models minimal information standard (PDX-MI).**

**S2 Table**. **Range of PAPP-A concentration (ng).**

Supplementary 1

|  | **PDX Model (PH)** | | | | | | | |
| --- | --- | --- | --- | --- | --- | --- | --- | --- |
|  | **271** | **358** | **471** | **006** | **231** | **112** | **386** | **450** |
| **Gender** | F | F | F | F | F | F | F | F |
| **Age** | 66 | 60 | 69 | 78 | 60 | 51 | 71 | 59 |
| **Diagnosis** | Ovarian Cancer | Ovarian Cancer | Ovarian Cancer | Ovarian  Cancer | Ovarian Cancer | Ovarian Cancer | Fallopian Tube Cancer | Ovarian Cancer |
| **Consent** | Academic | Academic | Academic | Academic | Academic | Academic | Academic | Academic |
| **Primary Tissue** | Ovary | Ovary | Ovary | Ovary | Ovary | Ovary | Fallopian Tube | Ovary |
| **Collection Site** | Primary | Primary | Primary | Primary | Metastasis | Primary | Metastasis | Primary |
| **Specimen collected** | Ovary | Ovary | Ovary | Ovary | Omentum | Ovary | Ovary | Ovary |
| **Histology** | Squamous/  Transitional | Serous | Clear Cell | Malignant Mixed Mullerian | Serous | Serous | Serous | Clear Cell |
| **Grade** | High | High | High | High | High | High | High | High |
| **Stage** | FIGO IV | FIGO IIIC | FIGO IIIC | FIGO IIIC | FIGO IIIC | FIGO IC | FIGO IIIB | FIGO IC |
| **Markers** | N/A | N/A | N/A | N/A | N/A | N/A | N/A |  |
| **Treatment** | Naïve | Naïve | Naïve | Naïve | Naïve | Naïve | Naïve | Naïve |
| **Mouse Strain** | SCID-bg | SCID-bg | SCID-bg | SCID-bg | SCID-bg | SCID-bg | SCID-bg | SCID-bg |
| **Mouse Humanized** | No | No | No | No | No | No | No | No |
| **Preparation** | Solid Tumor | Solid Tumor | Solid Tumor | Solid Tumor | Solid Tumor | Solid Tumor | Solid Tumor | Solid Tumor |
| **Injection site** | IP | IP | IP | IP | IP | IP | IP | IP |
| **Characterization** | Histology | Histology | Histology | Histology | Histology | Histology | Histology | Histology |
| **Negative murine/EBV** | Yes | Yes | Yes | Yes | Yes | Yes | Yes | Yes |
| **Passage** | P3 | P4 | P3 | P4 | P5 | P3 | P3 | P3 |

Supplementary 2. First-generation OC PDX models were screened for human PAPP-A mRNA using an ultrasensitive

| PDX MODEL | PAPP-A CONCENTRATION |
| --- | --- |
| 231 | 1.3200 |
| 534 | 0.7210 |
| 053 | 0.6700 |
| 271 | 0.5300 |
| 471 | 0.4450 |
| 061 | 0.3300 |
| 006 | 0.1800 |
| 358 | 0.1300 |
| 503 | 0.0650 |
| 526 | 0.0600 |
| 386 | 0.0300 |
| 467 | 0.0270 |
| 081 | 0.0270 |
| 384 | 0.0200 |
| 134 | 0.0180 |
| 112 | 0.0160 |
| 045 | 0.0160 |
| 450 | 0.0150 |
| 087 | Below the level of detection |
| 113 | Below the level of detection |

Supplementary 3


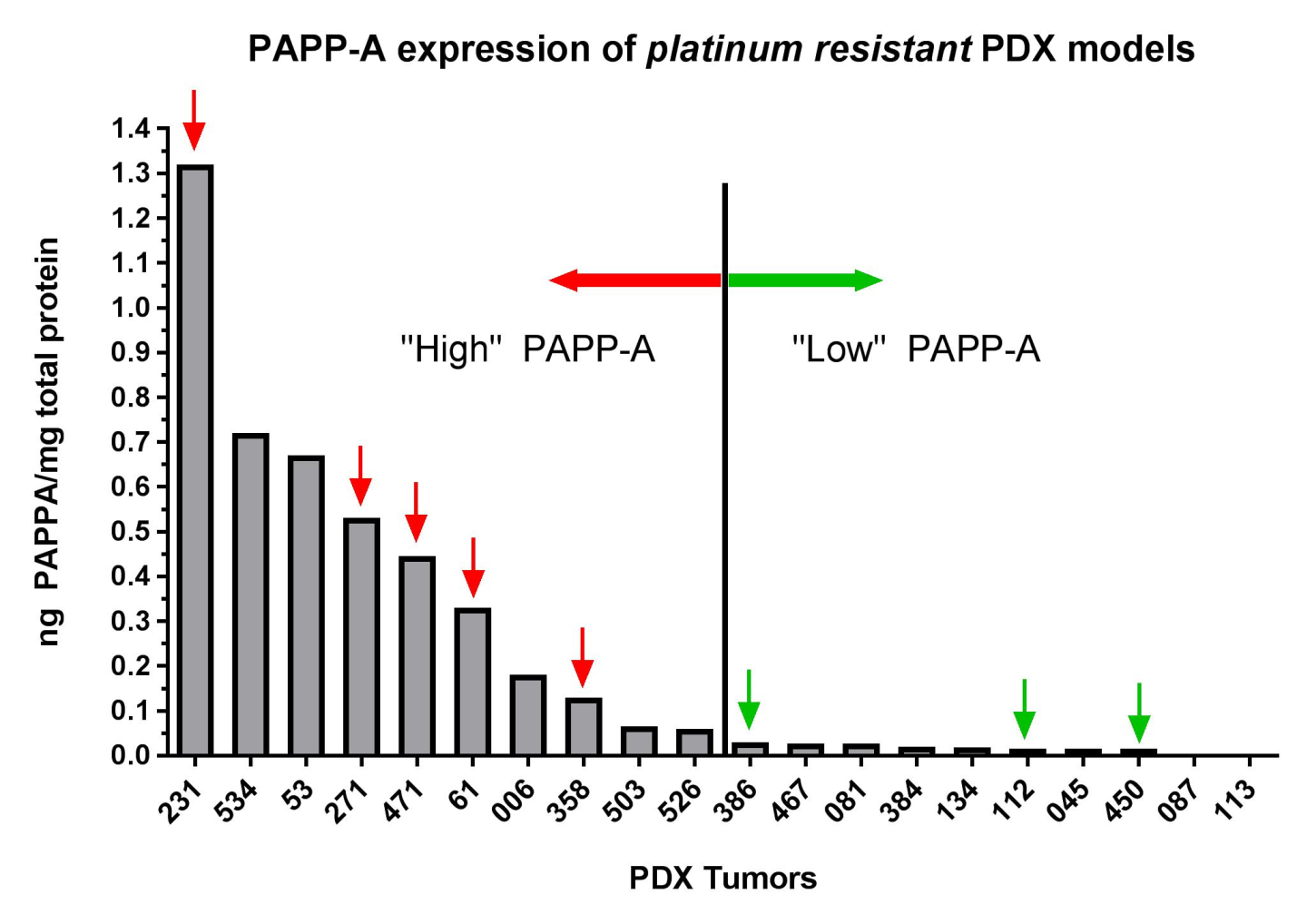


Supplementary 4


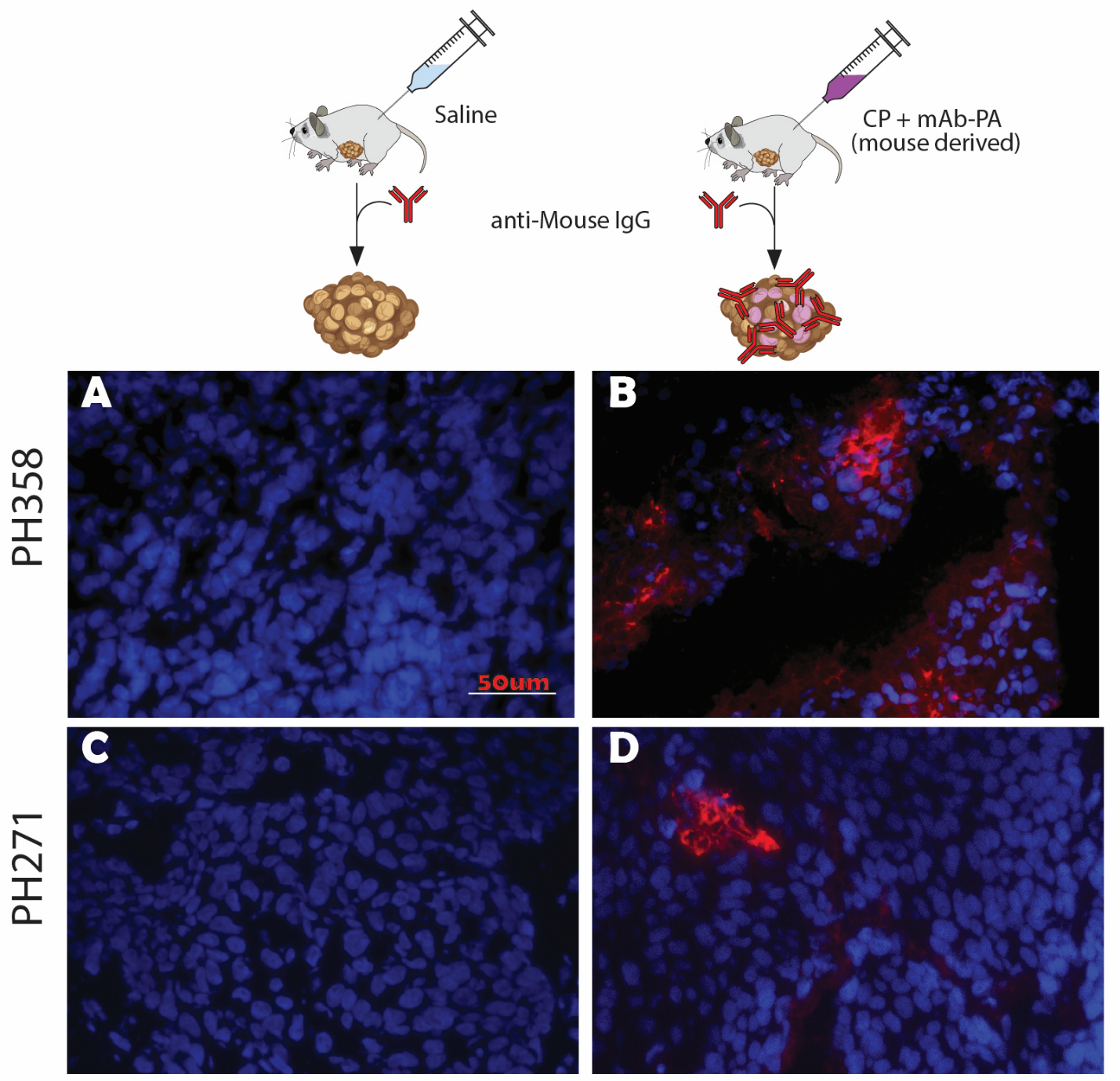


Supplementary 5


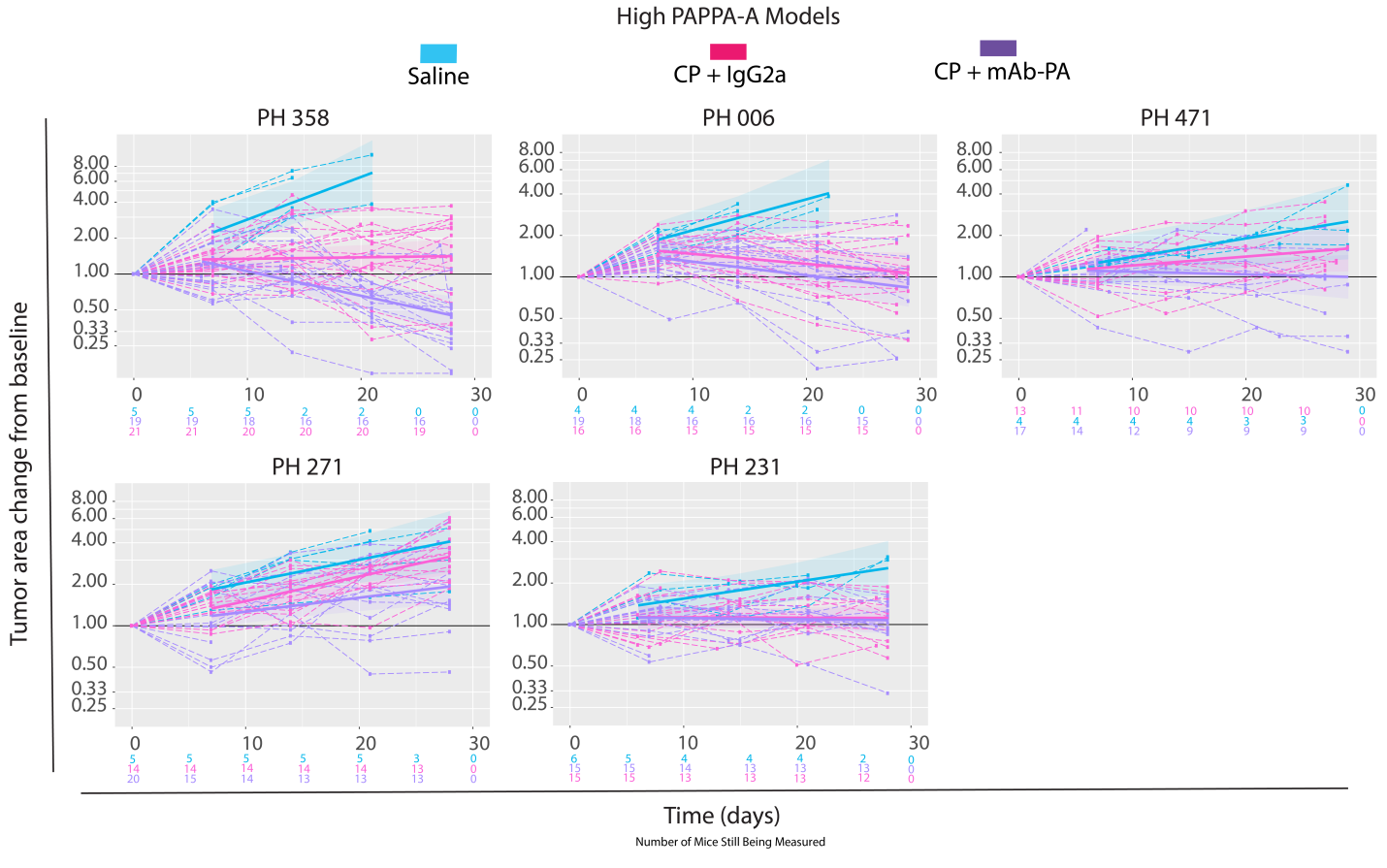

Supplement: S1 Supplementary statistical methods — Thus, exponential growth is assumed. Predictor variables included time, study arm, and the interaction between time and study arm. The time variable was centered for hypothesis testing, and thus can be interpreted as the overall average or an area under the curve[28–31]. The slope is interpreted as a rate of growth. The intercept and slope were specified as random effects with unstructured correlation, allowing per-mouse regression lines. Because of occasional differences in measurement intervals, a spatial power correlation structure was used, which assumes any two observations from the same mouse are correlated and that this correlation decreases exponentially with time between the observations. For visualization, model estimates with 95% confidence intervals were plotted for each treatment group and are displayed as shadows. A two degree of freedom test of coincident curves utilized data after baseline through day 28 to compare growth rates between treatment arms. The null hypothesis is that the growth curves are coincident, i.e., have the same intercept (mean) and slope. The alternative hypothesis is that the growth curves differ in intercept, slope or both. Average results are based on model predicted values. (DOCX) [file pone.0224564.s001.docx]
