# Supplementary figures and images for: Overcoming platinum resistance in ovarian cancer by targeting pregnancy-associated plasma protein-A
Source: PLoS One. 2019 Nov 21;14(11):e0224564. doi: 10.1371/journal.pone.0224564 (PMC6872139; doi:10.1371/journal.pone.0224564)

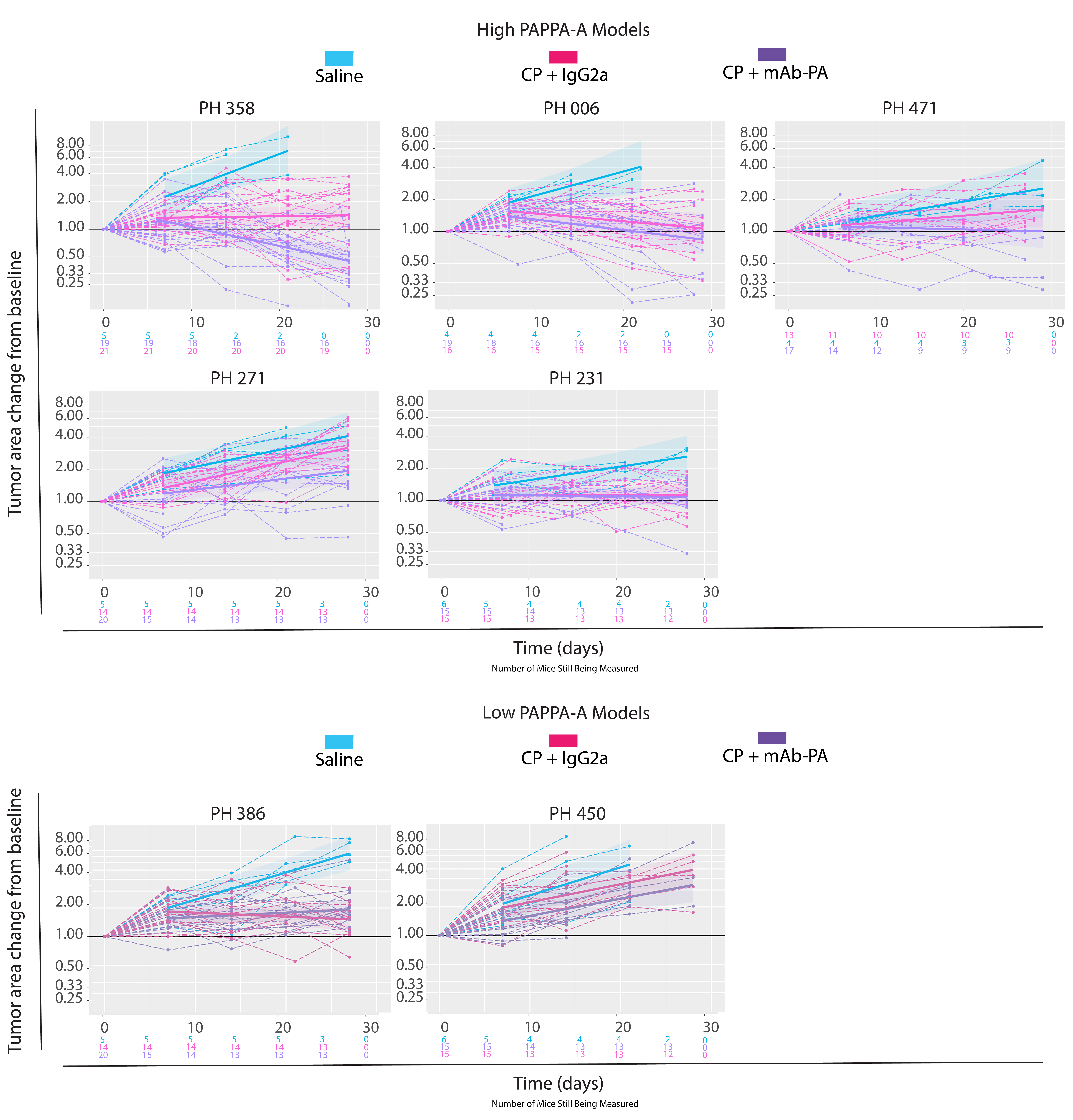

Supplement: S1 Fig — Dashed lines are individual mouse tumor area trajectories as a function of time on the fold change from baseline scale. Solid lines with shading are model predicted values with 95% confidence intervals. Numbers below the x-axis indicate number of mice still being followed at each time point for each treatment group. (TIF) [file pone.0224564.s002.tif]

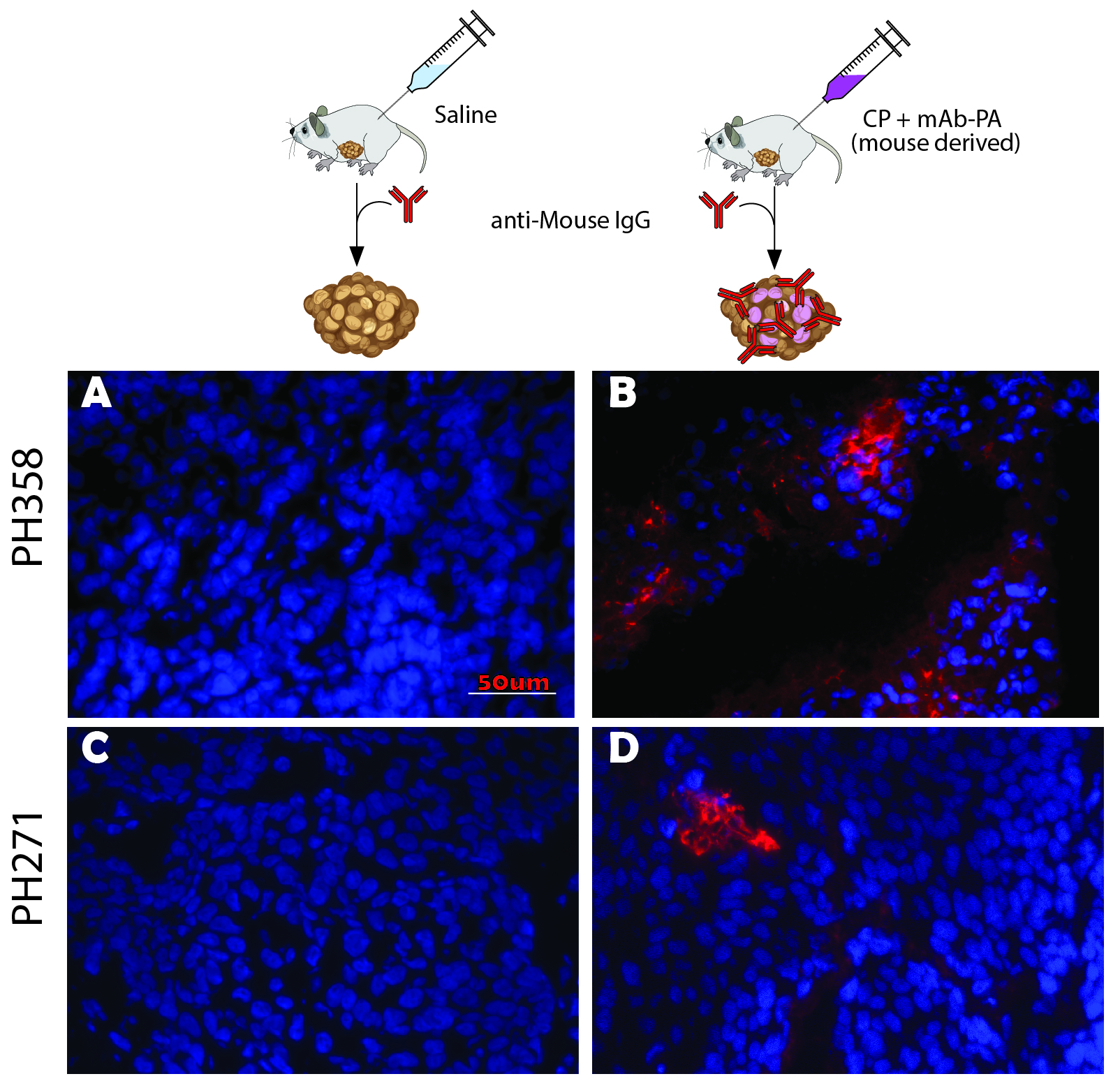

Supplement: S3 Fig — Post-treated samples from a saline control (left) and Carboplatin/Paclitaxel (CP) plus mAb-PA (right) were probed with a poly-clonal anti-mouse antibody to detect presence of mAb-PA or background mouse IgG. A high PAPP-A model (PH358), which regressed below baseline when treated with CP + mAb-PA, show no background mouse IgG [A] and positive staining (red) for mAb-PA intratumor penetration [B]. A similar pattern was observed with PH271 [C and D], which did regress below baseline when treated with CP + mAb-PA. Tumors treated with CP + IgG2a had similar immunofluorescent staining patterns to panels [B] and [D] (not shown). DAPI was used to stain nuclei (blue). (TIF) [file pone.0224564.s004.tif]
