# Supplementary material for: Overcoming platinum resistance in ovarian cancer by targeting pregnancy-associated plasma protein-A
Source: PLoS One. 2019 Nov 21;14(11):e0224564. doi: 10.1371/journal.pone.0224564 (PMC6872139; doi:10.1371/journal.pone.0224564)
Supplement: S1 Table — (DOCX) [file pone.0224564.s005.docx]

| **PDX Models (PH)** | | | | | | | |
| --- | --- | --- | --- | --- | --- | --- | --- |
|  | **271** | **358** | **471** | **006** | **231** | **386** | **450** |
| **Gender** | F | F | F | F | F | F | F |
| **Age** | 66 | 60 | 69 | 78 | 60 | 71 | 59 |
| **Diagnosis** | Ovarian Cancer | Ovarian Cancer | Ovarian Cancer | Ovarian  Cancer | Ovarian Cancer | Fallopian Tube Cancer | Ovarian Cancer |
| **Consent** | Academic | Academic | Academic | Academic | Academic | Academic | Academic |
| **Primary Tissue** | Ovary | Ovary | Ovary | Ovary | Ovary | Fallopian Tube | Ovary |
| **Collection Site** | Primary | Primary | Primary | Primary | Metastasis | Metastasis | Primary |
| **Specimen collected** | Ovary | Ovary | Ovary | Ovary | Omentum | Ovary | Ovary |
| **Histology** | Squamous/  Transitional | Serous | Clear Cell | Malignant Mixed Mullerian | Serous | Serous | Clear Cell |
| **Grade** | High | High | High | High | High | High | High |
| **Stage** | FIGO IV | FIGO IIIC | FIGO IIIC | FIGO IIIC | FIGO IIIC | FIGO IIIB | FIGO IC |
| **Markers** | N/A | N/A | N/A | N/A | N/A | N/A |  |
| **Treatment** | Naïve | Naïve | Naïve | Naïve | Naïve | Naïve | Naïve |
| **Mouse Strain** | SCID-bg | SCID-bg | SCID-bg | SCID-bg | SCID-bg | SCID-bg | SCID-bg |
| **Mouse Humanized** | No | No | No | No | No | No | No |
| **Preparation** | Solid Tumor | Solid Tumor | Solid Tumor | Solid Tumor | Solid Tumor | Solid Tumor | Solid Tumor |
| **Injection site** | IP | IP | IP | IP | IP | IP | IP |
| **Characterization** | Histology | Histology | Histology | Histology | Histology | Histology | Histology |
| **Negative murine/EBV** | Yes | Yes | Yes | Yes | Yes | Yes | Yes |
| **Passage** | P3 | P4 | P3 | P4 | P5 | P3 | P3 |
