# Supplementary material for: Overcoming platinum resistance in ovarian cancer by targeting pregnancy-associated plasma protein-A
Source: PLoS One. 2019 Nov 21;14(11):e0224564. doi: 10.1371/journal.pone.0224564 (PMC6872139; doi:10.1371/journal.pone.0224564)
Supplement: S2 Table — (DOCX) [file pone.0224564.s006.docx]

S2 Table. First-generation OC PDX models were screened for human PAPP-A mRNA using an ultrasensitive ELISA assay.

| PDX MODEL | PAPP-A CONCENTRATION |
| --- | --- |
| 231 | 1.3200 |
| 534 | 0.7210 |
| 053 | 0.6700 |
| 271 | 0.5300 |
| 471 | 0.4450 |
| 061 | 0.3300 |
| 006 | 0.1800 |
| 358 | 0.1300 |
| 503 | 0.0650 |
| 526 | 0.0600 |
| 386 | 0.0300 |
| 467 | 0.0270 |
| 081 | 0.0270 |
| 384 | 0.0200 |
| 134 | 0.0180 |
| 045 | 0.0160 |
| 450 | 0.0150 |
| 087 | Below the level of detection |
| 113 | Below the level of detection |
